# A world map of evidence-based medicine: Density equalizing mapping of the Cochrane database of systematic reviews

**DOI:** 10.1371/journal.pone.0226305

**Published:** 2019-12-13

**Authors:** David A. Groneberg, Stefan Rolle, Michael H. K. Bendels, Doris Klingelhöfer, Norman Schöffel, Jan Bauer, Dörthe Brüggmann

**Affiliations:** The Institute of Occupational, Social and Environmental Medicine, Goethe University Frankfurt, Frankfurt, Germany; University of Mississippi Medical Center, UNITED STATES

## Abstract

Systematic reviews represent the core and backbone of evidence-based medicine (EBM) strategies in all fields of medicine. In order to depict a first global sketch of the international efforts in the Cochrane database systematic reviews (CDSR), we analyzed the systematic reviews of the Cochrane database. Our global maps of systematic reviewing offer intriguing structural insights into the world of EBM strategies. They demonstrate that for the CDSR, the UK and Commonwealth countries take the lead position. Since patients, care providers and health systems all over the world benefit from systematic reviewing, institutions in other countries should increase their commitment.

## Background

Systematic reviews constitute the backbone of evidence-based medicine (EBM) in all fields of medicine. Among the different study types that rely upon evidence-based medicine, the Cochrane Database of Systematic Reviews (CDSR) plays a key role in this important field of medicine [1]. Besides the benefits of CDSRs, there has to be some critical issues to be raised. To be named are the lack of comparability, the risks of bias, indirectness, imprecision, and inconsistency [2]. While the CDSR has nearly published 10,000 systematic reviews so far, the underlying architecture has not been analyzed so far using advanced scientometric tools. In order to address this issue and to depict a first sketch of the global landscape of systematic reviews, we used the NewQIS platform, which was established in 2007/8 [3, 4]. So far, about 50 different biomedical entities were analyzed on the basis of NewQIS ranging from infectious diseases such as Ebola to cancer or even health care policy issues [5–7]. To generate maps of systematic reviewing, the Web of Science (WoS) Core Collection database was used since it also allows citation analysis and density equalizing projections, established by Gastner and Newman [8]. All CDSR articles were analyzed and reviewed with a focus on the question arises who dominates systematic reviewing processes from a global viewpoint. Since US institutions are known to play a prominent role in almost every area of medicine [9], it was hypothesized that they also dominate systematic reviewing in the CDSR.

## Methods

### NewQIS protocol

We used the previously established NewQIS platform to analyze all CDSR publications present in the Web of Science (WoS) Core Collection database. The WoS was chosen because of the ability to analyze citations.

### Search protocol

We identified all relevant articles by searching the WoS for the following string: PUBLICATION NAME: Cochrane Database of Systematic Reviews, Refined by DOCUMENT TYPES: review or article, Timespan: all years.

### Density-equalizing mapping

Density-equalizing mapping projections (DEMPs) were used: In brief, DEMPs algorithms based on Gastner and Newman’s algorithm [8] were used to re-size country territories with regard to the following variables: 1) number of published items of each country, 2) the number of institutions in each country, 3) the number of citations for each country, 4) the country-specific modified Hirsch index. The original Hirsch (H)-index for authors was defined by Jorge Hirsch, representing a quantifying index (h): h is the maximum number from the total number of articles written by a given author where each one of these h articles have been at least h times cited. Presently, a modified h index was used to quantify citations of countries as previously performed in the NewQIS studies.

### Network analysis

A matrix with all participant countries was computed and transformed into a vector graphic. The vector thickness illustrates the numbers of co-operations between two countries. A threshold of at least two collaborations was set.

### Contextual factors

Socio-economic data was obtained from the World Factbook [10]. The values of the Gross Domestic Product (GDP) per publishing country were related to publication activities. The number of researchers in full time equivalent (FTE) per mill. inhabitants was extracted from the UNESCO database [11]. Consequently, these number were also set in relation to the number of CDSRs.

## Results

### General activity

**Fig 1:**
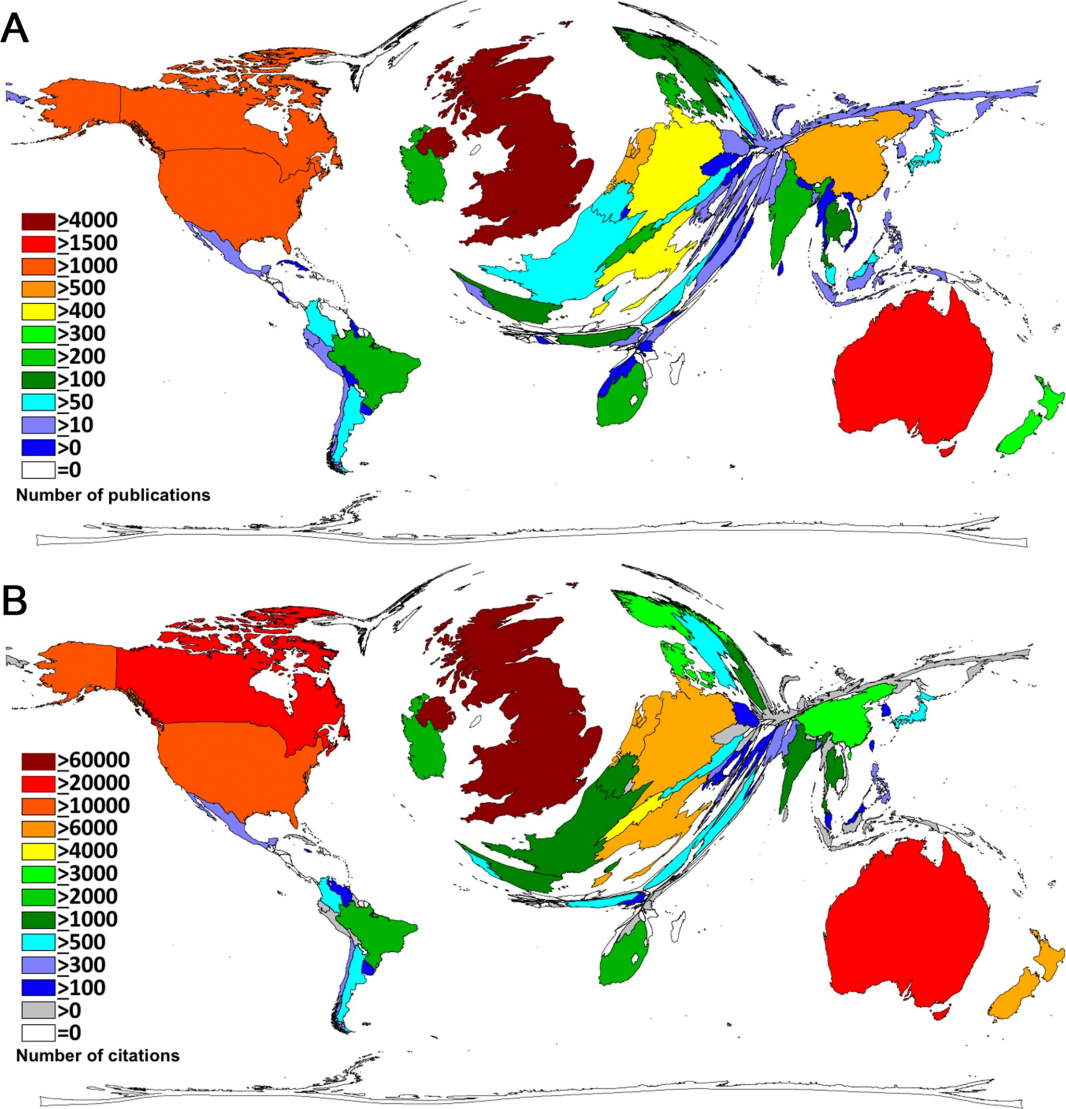
World map of Cochrane database of systematic review activity. A) Density equalizing map projection of the number of articles per country. B) Density equalizing map projection of the number of citations.

A total of n = 9,765 CDSR articles were identified using the above described search methodology. When these publications were ranked for the highest country activities, the UK was ranked no. 1 with a total of n = 4,261 articles. It was followed by Australia with a total of n = 1.596. At third position, the USA was listed with n = 1,309 articles, followed by Canada (n = 1,094). Active countries were also the Netherlands (n = 699), China (n = 576), Italy (n = 466), Germany (n = 423), New Zealand (n = 376), Brazil (n = 292), Switzerland (n = 260), Denmark (n = 223), India (n = 208), and Ireland (n = 201). When all countries are analyzed using density equalizing mapping, a global landscape appears, which is largely distorted towards Europe with the UK, Northern America with Canada and the USA, and Australia (Fig 1A). While Africa nearly disappears, Eastern Asia is visible due to Chinese contributions (n = 576).

### Quality parameters

**Fig 2:**
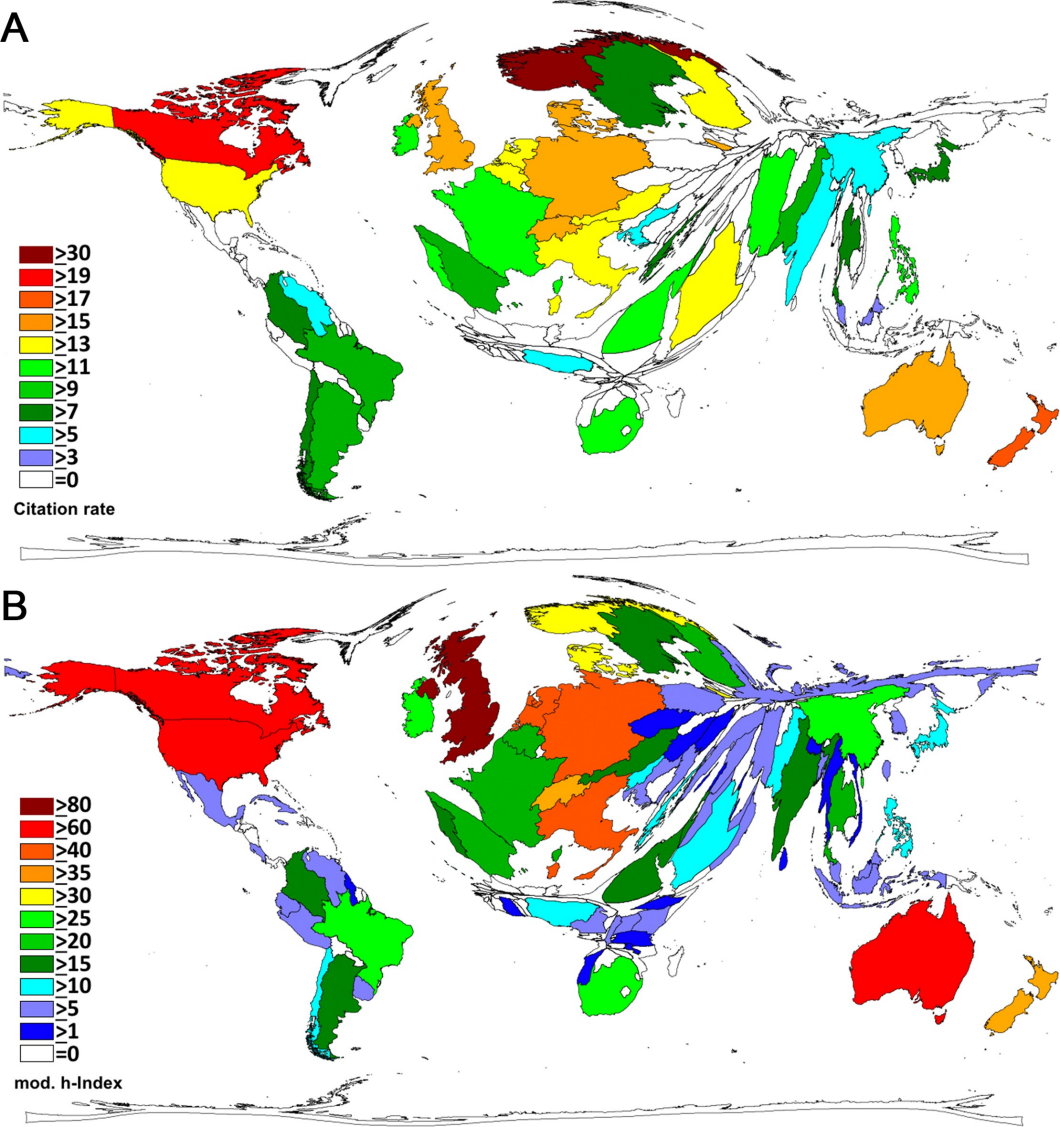
World map of Cochrane database of systematic review activity. A) Density equalizing map projection of the citation rates. B) Density equalizing map projection of the country-specific h-indices.

To approach (semi-)qualitative aspects of CDSR research, citation analysis was performed using a variety of benchmarks. In the absolute citation ranking, the UK was ranked no.1 (c = 63,974 citations), followed by Australia (c = 25,894), Canada (c = 21,213), the US (c = 18,971), the Netherlands (c = 9,559), New Zealand (c = 6,884), Italy (n = 6,602), Germany (c = 6,587), Switzerland (c = 4,261), and Norway (c = 3,566), China (c = 3,555), and Denmark (c = 3,547). Density equalizing mapping leads to a distortion of the world map (Fig 1B) that largely follows the total publication activity mapping. The citation rate analysis shows a different ranking: The highest number of citations per review (cr) is found for Norway (cr = 32.41), followed by Canada (cr = 19.39), and New Zealand (cr = 18.3), underlying a threshold of at least 30 CDSRs. The UK has a citation rate of about cr = 15 and the USA of cr = 14.5 (Fig 2). The country-specific h-index (hI) analysis lists the UK at hI = 89, followed by Australia (hI = 69), Canada (hI = 66) and the US (hI = 60) (Fig 2).

### International networking

**Fig 3:**
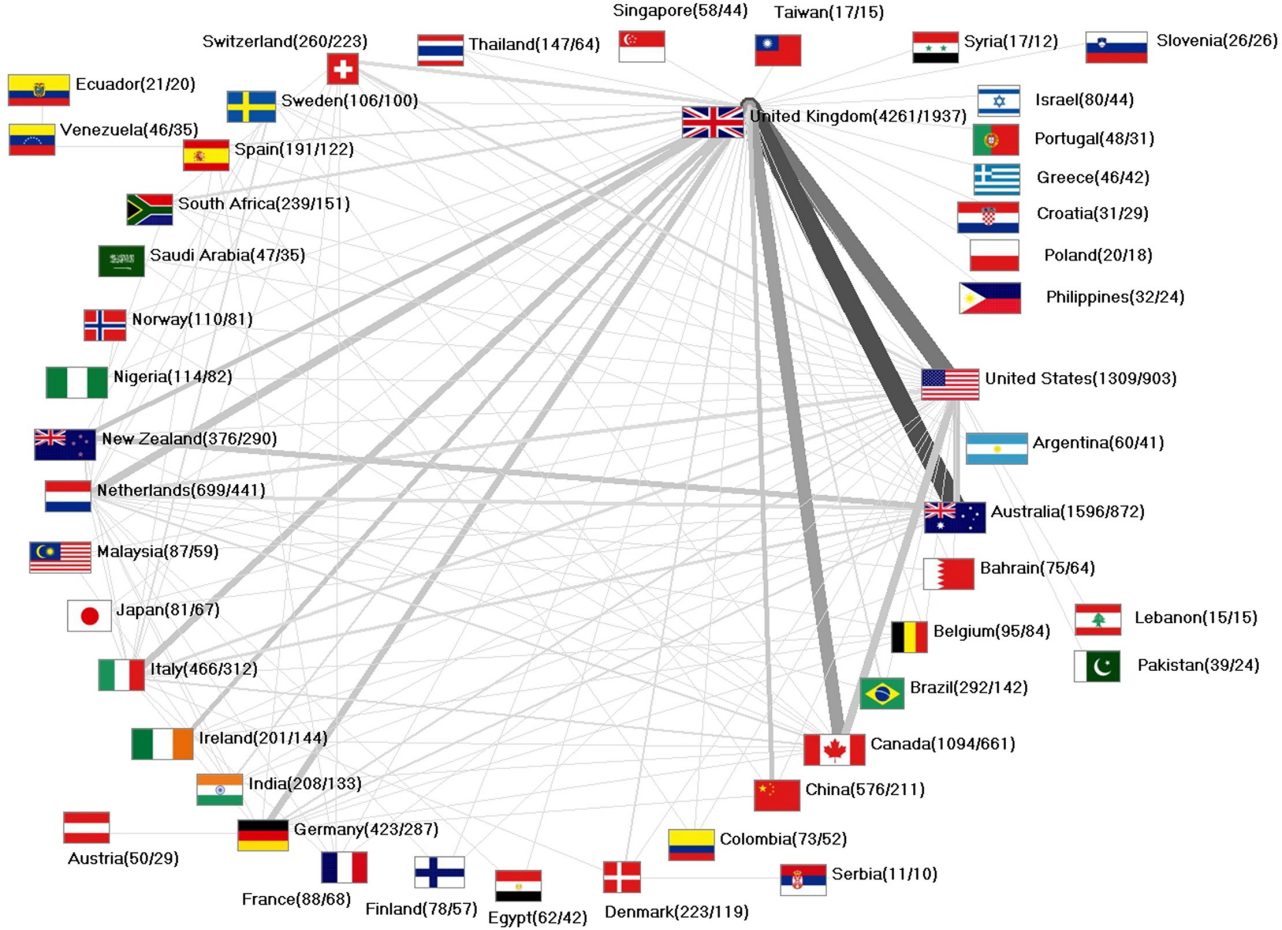
International network–number of multinational cooperation articles.

Networking analysis of all articles shows that there are strong bonds between the UK (1,937 out of all 4,261 UK reviews are performed as international collaborations) and Australia, the US and Canada. Interestingly, the US has a much higher percentage of collaborative articles (903/1,309 = 69%) than the UK (45.5%) or Australia (54.6%). There are also countries such as Sweden (94.3%), which publish nearly all reviews within an international collaboration (Fig 3).

### Contextual factors

When the economic strength of the highly industrialized countries is related to the systematic reviewing activities of these countries. The following data are obtained (Table 1): New Zealand publishes a calculated 2.151 reviews per 1000 billion US$ Gross Domestic Product (GDP), the UK 1,528 reviews per 1000 billion US$ GDP and Australia 1,342. By comparison, the USA publishes about 71 systematic reviews per 1000 billion US$ GDP, Germany 106 and China 27 systematic reviews per 1000 billion US$ GDP, respectively. Secondly, the number of reviews can also be related to the number of researchers (FTE) per mill. inhabitants (researchers per capita): Here, the UK leads with 0.97 reviews per researchers per capita, followed by India with 0.96 reviews per researchers per capita, and Colombia with 0.83 reviews per researchers per capita. China has 0.47 reviews per researchers per capita ranking 10^th.^ The US is ranked 12^th^ with 0.31 reviews per researchers per capita.

**Table 1:** Cochrane database of systematic reviews–socio-economic features. GDP Gross Domestic Product; bn billion; USD United States Dollar; HI high-income country; UMI upper-middle-income country; LMI lower-middle-income country, FTE full time equivalents. Sources: GDP (bn USD) [10], Number of Researcher (FTE) per mill. inhabitants [11],–no data available.

Country	No. of articles	GDP (bn USD)	Articles/GDP (1000 bn USD)	Rank	Researchers (FTE) per mill. inhabitants	Articles/ Researchers (FTE) per mill. inhabitants	Rank
New Zealand	376	174.8	2151.03	HI1	4052.42	0.0928	HI8
United Kingdom	4261	2788	1528.34	HI2	4376.96	0.9735	HI1
Australia	1596	1189	1342.30	HI3	–	–	–
Bahrain	75	66.37	1130.03	HI4	368.90	0.2033	HI5
Denmark	223	264.8	842.15	HI5	7896.85	0.0282	HI14
Netherlands	699	865.9	807.25	HI6	5007.06	0.1396	HI7
Canada	1094	1674	653.52	HI7	4274.70	0.2559	HI4
Ireland	201	324.3	619.80	HI8	4107.60	0.0489	HI13
Switzerland	260	494.3	526.00	HI9	5257.36	0.0495	HI12
Croatia	31	94.24	328.95	HI10	1865.44	0.0166	HI18
Finland	78	239.2	326.09	HI11	6707.49	0.0116	HI22
South Africa	239	736.3	324.60	HI12	493.72	0.4841	HI2
Norway	110	364.7	301.62	HI13	6477.76	0.0170	HI17
Israel	80	297	269.36	HI14	8250.47	0.0097	HI24
Sweden	106	498.1	212.81	HI15	7592.50	0.0140	HI21
Italy	466	2221	209.82	HI16	2294.55	0.2031	HI6
Belgium	95	508.6	186.79	HI17	4941.98	0.0192	HI16
Portugal	48	297.1	161.56	HI18	4290.82	0.0112	HI23
Greece	46	290.5	158.35	HI19	3152.84	0.0146	HI20
Thailand	147	1161	126.61	UMI1	1210.35	0.1215	UMI5
Austria	50	415.9	120.22	HI20	5157.52	0.0097	HI25
Singapore	58	486.9	119.12	HI21	6729.68	0.0086	HI26
Spain	191	1690	113.02	HI22	2873.41	0.0665	HI11
Germany	423	3979	106.31	HI23	5036.18	0.0840	HI9
Colombia	73	690.4	105.74	UMI2	88.48	0.8250	UMI1
Nigeria	114	1089	104.68	LMI1	–	–	–
Malaysia	87	863.8	100.72	UMI3	2357.92	0.0369	UMI8
Venezuela	46	468.6	98.16	UMI4	283.93	0.1620	UMI4
Chile	41	436.1	94.02	HI24	502.10	0.0817	HI10
Brazil	292	3135	93.14	UMI5	881.38	0.3313	UMI3
United States	1309	18560	70.53	HI25	4256.29	0.3075	HI3
Argentina	60	879.4	68.23	UMI6	1232.60	0.0487	UMI7
Egypt	62	1105	56.11	LMI2	669.39	0.0926	LMI4
Philippines	32	801.9	39.91	LMI3	187.66	0.1705	LMI2
Pakistan	39	988.2	39.47	LMI4	354.13	0.1101	LMI3
France	88	2737	32.15	HI26	4441.07	0.0198	HI15
Saudi Arabia	47	1731	27.15	HI27	–	–	–
China	576	21270	27.08	UMI7	1234.78	0.4665	UMI2
Iran	35	1459	23.99	UMI8	671.02	0.0522	UMI6
India	208	8721	23.85	LMI5	216.18	0.9622	LMI1
Japan	81	4932	16.42	HI28	5304.90	0.0153	HI19

**Fig 4:**
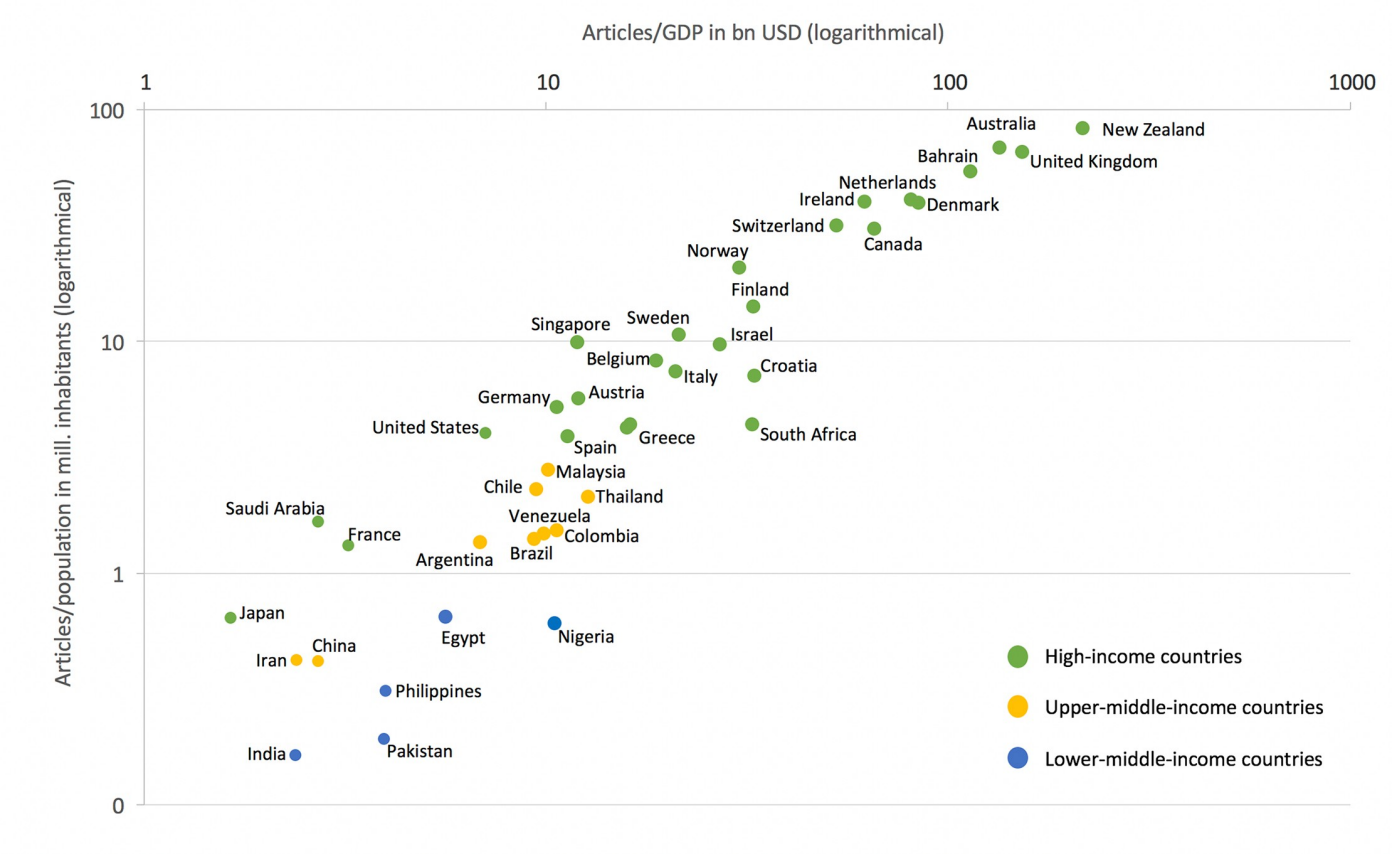
Countries’ distribution pattern regarding the number of reviews per inhabitants in mill. and the number of reviews per GDP (gross domestic product) per 1000 bn US-dollars.

In consideration of the socio-economic parameters GDP and total population of the publishing countries a clear economic related distribution appears (Fig 4).

## Discussion

The area of evidence-based medicine has increasing importance in all areas of medicine. We analyzed the Cochrane database systematic reviews using the NewQIS platform and density equalizing approaches and found striking contrasts to the usual global research patterns: A large study analyzed a number of 5,527,558 publications and showed the usually clear dominance in biomedicine of the USA. The USA was by far the most productive country with nearly 1.9 million reviews. Japan ranked second with 573,473 reviews. The third most active country was Germany (n = 444,775), followed by the UK (n = 415,499). When oncology is focused, a similar lead position for the US is present in numerous cancers including ovarian cancer [6], breast cancer [12], pancreatic cancer [13] or laryngeal cancer [14], as previously demonstrated. In contrast, our findings show the UK being by far the leading country within the area of systematic reviews. Thus, our primary hypothesis needs to be rejected. Out of the 9,765 CDSRs included in our analysis, 4,261 originated from the UK and just 1,309 articles from the USA. A further interesting finding is that China appears to be strong with 576 reviews, in contrast with Japan with just 81 reviews. Even Malaysia (n = 87) appeared to be more active in this area of the world. Noteworthy, Japanese research activity is currently regarded to be decreasing in contrast to China [15]. As reported by N. Philips [16], Japan's Council for Science, Technology and Innovation conceded in on its Fifth Science and Technology Basic Plan (2015) that its world standing in science and technology was falling.

The total number of systematic reviews might represent a good marker for overall activities in the field of clinical medicine, in which evidence based diagnosis and treatment are of great importance [17]–but how meaningful are they? This can be approximated by citation analysis. Total numbers of citations largely follow the total numbers of publications. More detailed information can be obtained by assessing the citation rates and country-specific h-indices. Here, the highest citation rate is present for Norway, followed by Canada and New Zealand. A similar pattern with Scandinavian countries in the leading position has also been discovered in other studies [18]. To the same extend, Scandinavian countries such as Sweden seem to have a very high degree of collaborative research–we found that 94.3% of all Swedish Cochrane articles were published as international collaboration. It is noteworthy that the US also have a relatively high proportion of international collaborations (69%).

Since systematic reviews constitute the backbone of evidence-based medicine, highly industrialized countries should allocate reasonable resources to the advancement of science in this field. Our calculations show that the Commonwealth countries New Zealand (calculated 2.151 reviews per 1000 billion US$ GDP), the UK (1.528 reviews per 1000 billion US$ GDP). and Australia (1.342 reviews per 1000 billion US$ GDP) are very active with the US just publishes about 71 systematic reviews per 1000 billion US$ GDP. Again, Japan has a very poor rate with 16.42 systematic reviews per 1000 billion US$ GDP. The UK also lead when the global Cochrane database systematic review activity is related to the countries´ number of researchers per capita. With India (n = 208) and Colombia (n = 73), two countries that have no high-income status are ranked next. The most collaborations of the Indian reviews were worked out with the UK. Colombia mostly worked together with Spain. Both connections are certainly caused by the historical connection and the common language.

What is the reason for this imbalance within highly industrialized countries such as the USA and highly industrialized Commonwealth countries such as the UK or Australia? One reason might be found in the history of the Cochrane database of systematic reviews. Cochrane is named in honor of Professor Archibald Cochrane, a Scottish physician who pioneered the field of evidence-based medicine. His visions were the founding principles that led to the opening of the first Cochrane Centre in Oxford, UK in 1992 and the founding of the Cochrane Collaboration in 1993 [19]. A second reason might be the geographical distribution of Cochrane Review Group editorial bases and the British origin of most of the editors. However, countries without review group editorial bases in countries such as China, Switzerland, Brazil, Ireland, or India are much more active than countries with bases in countries such as France or Portugal.

## Conclusions

The established global maps in this study offer intriguing insights into the world of Cochrane systematic reviews that form the basis of evidence-based medicine. They clearly show that the UK and Commonwealth countries take the lead position in this extremely important field of medicine. Patients, care providers and health systems all over the world benefit from this commitment. With regard to the unusual *non-leading* role of the US and the great potential of US institutions, clinicians and scientists from the US should foster their activities in this field. Future studies using novel approaches might also assess gender distribution of Cochrane authors [20, 21].
